# Removal of Mercury (II) by EDTA-Functionalized Magnetic CoFe_2_O_4_@SiO_2_ Nanomaterial with Core-Shell Structure

**DOI:** 10.3390/nano9111532

**Published:** 2019-10-29

**Authors:** Kai Xia, Yongfu Guo, Qijun Shao, Qu Zan, Renbi Bai

**Affiliations:** 1 Center for Separation and Purification Materials &Technologies, Suzhou University of Science and Technology, Suzhou 215009, Chinaceebairb@live.com (R.B.); 2 Jiangsu Provincial Key Laboratory of Environmental Science and Engineering, Suzhou University of Science and Technology, Suzhou 215009, China; quzan@sjtu.edu.cn; 3 Department of Fundamental Courses, Wuxi Institute of Technology, Wuxi 214121, China; 4 School of Environmental Science and Engineering, Shanghai Jiao Tong University, Shanghai 200240, China

**Keywords:** magnetic material, adsorption, heavy metal, mercury, EDTA functionalization

## Abstract

In order to reduce the difficulty and risk of operation, decrease the preparation time and improve the adsorption performance of magnetic nano-silicon adsorbent with core-shell structure, a carboxylated CoFe_2_O_4_@SiO_2_ was prepared by EDTA-functionalized method using a safe, mild and simple hydrothermal method. The results show that the prepared material of CoFe_2_O_4_@SiO_2_-EDTA has a maximum adsorption capacity of 103.3 mg/g for mercury ions (Hg(II)) at pH = 7. The adsorption process of Hg(II) is a chemical reaction involving chelation and single-layer adsorption, and follows the pseudo-second-order kinetic and Langmuir adsorption isotherm models. Moreover, the removal of Hg(II) is a spontaneous and exothermic reaction. The material characterization, before and after adsorption, shows that CoFe_2_O_4_@SiO_2_-EDTA has excellent recyclability, hydrothermal stability and fully biodegradable properties. To summarize, it is a potential adsorption material for removing heavy metals from aqueous solutions in practical applications.

## 1. Introduction

Currently, heavy metals, such as mercury, lead and copper, have extensively infiltrated natural water bodies that are becoming increasingly polluted [1]. Industrial wastewater generated in coal combustion and other industrial activities poses a threat to human health through bio-enrichment of aquatic organisms and sewage irrigation [2,3]. Heavy metals are easily accumulated in the food chain due to their non-degradable properties. Mercury pollution, in particular, is extremely dangerous because of its persistence, fluidity, high bioaccumulation and uncontrollability [4]. Moreover, it seriously jeopardizes the ecological environment, as well as human life and health [5]. Therefore, the World Health Organization (WHO) and the Environmental Protection Agency (EPA) have listed mercury as one of the most toxic elements of heavy-metal pollution [6]. The limits for mercury ions allowed in drinking water and surface water discharge standards are 2 and 10 µg/L, respectively. Mercury pollution is caused by industries, such as the chlor-alkali industry, plastics industry, dye chemicals industry and electronics industry, as well as activities like amalgamation of alfalfa [7]. Note that mercury exists in various chemical forms, including elemental mercury (Hg^0^), methylmercury (CH_3_Hg^+^) and inorganic mercury (Hg(II)) in environment. Therefore, the economic and efficient removal of mercury from water bodies in environmental field has become a top priority.

Recently, many studies have focused on the development of effective and inexpensive methods, such as reduction, precipitation, ion exchange, reverse osmosis, adsorption and membrane separation [8,9,10,11]. Among these methods, the adsorption method has the advantages of high efficiency, economy, flexibility and easy operation. Moreover, it has been widely used as one of the most effective techniques for removing heavy metal ions. Furthermore, considerable attention has been focused on the development of adsorbents such as activated carbon [12], carbon nanotubes [13], ion exchange resins [14], chitosan [15] and graphene [16]. Among these absorbents, silica gel is a very popular inorganic porous polymer that has high pore connectivity, large pore volume and high surface area; therefore, it is one of the potential materials for adsorbing heavy metals [17,18]. The surface of silica gel contains a large amount of silanol groups (Si–OH) so that various functional groups can be grafted on it. Note that carboxyl groups (–COOH) is a common functional group that can adsorb mercury ions (Hg^2+^) owing to its good coordination with heavy metal ions. Commonly grafted carboxyl functional groups are obtained using sodium alginate [19], humic acid [20] and atom transfer radical polymerization by grafting with polyacrylic acid [21,22]. Furthermore, in situ introduction technology has been used to complete the modification of the carboxyl group [23]. Most carboxyl modification methods are tedious and complicated. It is typically carried out with toxic, harmful or hazardous solvents (triethylamine, thionyl chloride, methacrylic acid, etc.) as reaction medium [21]. Moreover, both the adsorption effect after carboxyl modification and the stability of the synthetic material are weak. Furthermore, conventional non-magnetic adsorption materials exhibit weak solid–liquid separation after adsorption, which considerably limits their application in water treatment. To tackle this limitation, researchers are now focusing on magnetic materials, which are considered to be green (environmentally friendly) materials.

When the particle diameter (d) is smaller, the specific surface area is higher, which promotes the diffusion of particles in the solution, therefore, it is preferred to use a small size adsorbent [24]. The preparation of iron-based materials (such as CoFe_2_O_4_) in particular is simple and inexpensive, and it is not necessary to carry out the reaction process under nitrogen environment compared to the preparation process of MnFe_2_O_4_ [25,26,27] and F_3_O_4_ [28]. The number of hydroxyl groups (M–OH) on the surface of CoFe_2_O_4_ (38.1%) was higher than that of Fe_3_O_4_ (25.4%). the amount of M–OH has an important influence on the performance of the adsorbent, and the material can easy be modified with the more M–OH and CoFe_2_O_4_ (46.99 emu/g) has stronger magnetic properties than MnFe_2_O_4_ (32.0 emu/g) [29]. Moreover, most importantly, magnetic adsorbents are easily separated from water under the action of an external magnetic field.

CoFe_2_O_4_ also has disadvantages that cannot be overcome by itself, such as low toxicity, corrosion under acidic conditions, etc., and it is generally possible to improve its acid resistance and solve the problem of low toxicity by forming a silica layer. In this study, a magnetic core of CoFe_2_O_4_ was used as adsorbent carrier, which was coated with silica to form a magnetic matrix material with a core-shell structure (CoFe_2_O_4_@SiO_2_) [30,31]. The combination of magnetic material in a silica shell not only improves the specific surface area of the adsorbent material, but also enhances the acid resistance of the magnetic nanomaterial. Simultaneously, to improve the above-mentioned defect of carboxyl modification, ethylene diaminetetraacetic acid dianhydride (EDTA) was employed as a chelating agent to introduce oxygen-containing functional groups. EDTA, which has a strong stabilizing effect, excellent reproducibility and fully biodegradable properties, forms a very strong complex with metal ions [32]. EDTA is used to bind metal ions in the practice of chelation therapy, for example, for treating mercury and lead poisoning [33]. Compared to general carboxyl materials, EDTA has additional carboxyl groups, and the adsorption effect of chelation is better than that of traditional chemical. Moreover, the method used here has the advantages of reducing the solvent’s toxicity, improving the safety of the experiment and controlling the reaction at room temperature and pressure conditions, thus considerably improving the grafting rate and simplifying the reaction conditions. Finally, the adsorbent of CoFe_2_O_4_@SiO_2_ modified by EDTA (CoFe_2_O_4_@SiO_2_-EDTA) was used to remove mercury ion (Hg(II)) from an aqueous solution. Finally, the adsorption kinetics, thermodynamics, regeneration and mechanism were all examined.

## 2. Materials and Methods

### 2.1. Materials

Ethylene glycol (GC, >99 wt%) was purchased from Aladdin (China). Cobalt chloride (CoCl_2_·6H_2_O), iron acetylacetonate, polyethylene glycol, ammonia water (25–28 wt%), tetraethyl silicate (98 wt%), 3-aminopropyltriethoxysilane (APTES) and ferric chloride hexahydrate were purchased from Macleans (China). Ethylene diaminetetraacetic acid dianhydride (EDTA anhydride), absolute ethanol (C_2_H_5_OH), acetic acid (CH_3_COOH), hydrochloric acid (HCl), sodium hydroxide (NaOH) and nitric acid (HNO_3_) were purchased from Sinopharm Chemical Reagent Co., Ltd. (Shanghai, China). These reagents were all analytical reagents.

### 2.2. Preparation of CoFe_2_O_4_@SiO_2_

CoFe_2_O_4_ was prepared using hydrothermal synthesis. Briefly, 2.379 g of CoCl_2_·6H_2_O, 7.0634 g of Fe(acac)_3_, 2.0 g of polyethylene glycol and 8.679 g of CH_3_COONa were mixed in 120 mL of ethylene glycol and continuously stirred in a water bath at 313 K for 30 min. Then, the reaction was kept at 473 K for 16 h. The material was washed with ethanol and pure water and dried at 333 K. Next, 1.0 g of the as-prepared CoFe_2_O_4_ was poured into pure water and sonicated for 20 min. The magnetic solution was continued to be stirred for 30 min and heated to 353 K in a water bath. Subsequently, 2 mL of ammonia water and 2 mL of tetraethyl orthosilicate (TEOS) were added and then the reaction was executed at 353 K for 3 h. Finally, the solution was magnetically separated, washed with pure water, and dried at 333 K.

### 2.3. Preparation of CoFe_2_O_4_@SiO_2_-EDTA

**Figure 1:**
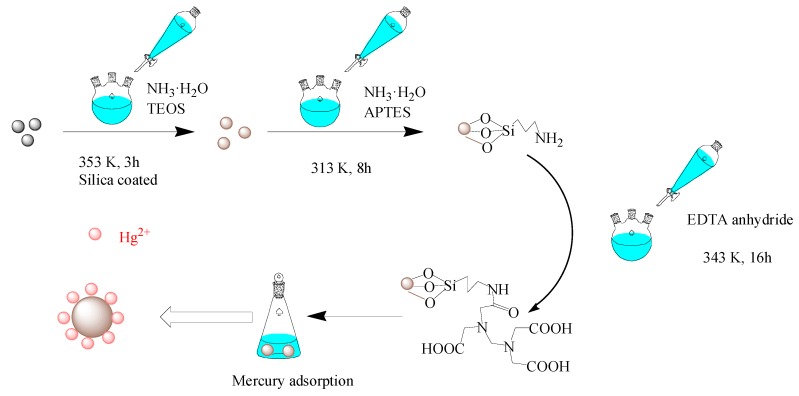
Synthesized schematic diagram of CoFe_2_O_4_@SiO_2_-EDTA in this experiment.

A total of 0.6 g of the as-prepared CoFe_2_O_4_@SiO_2_ was added into a 150 mL solution of ethanol and pure water (4:1) at 313 K for 0.5 h. Then 2 mL of ammonia water was added to adjust the pH to 9, and then 2 mL of TEOS was slowly added. After 1 h, 2 mL APTES was slowly added and the mixture was continued stirring for 8 h. The solution was then magnetically separated, washed with pure water and dried at 333 K. In this method, CoFe_2_O_4_@SiO_2_-NH_2_ was successfully prepared. Then, the amino group bounded to the surface of the silica was reacted with 9 mmol of EDTA anhydride in 160 mL of ethanol and acetic acid (1:1) at 343 K for 16 h. Then the magnetic separation material was washed with pure water and dried it at 333 K to obtain CoFe_2_O_4_@SiO_2_-EDTA. Please see the synthesized schematic diagram of CoFe_2_O_4_@SiO_2_-EDTA in Figure 1.

### 2.4. Sample Characterization

The appearance, structure and size of the material were characterized using transmission electron microscopy (TEM, Tecnai G2 F30, Hillsboro, OR, USA) and scanning electron microscopy (SEM, ZEISS-SUPRA 55, Jeona, Germany). X-ray diffraction (XRD, D8 Advance, Bruker, Karlsruhe, Germany) was used to analyse the crystal structure of the material. The functional groups in the material were characterized using Fourier transform infrared spectroscopy (FT-IR, Nicolet 6700, Thermo Scientific, Waltham, MA, USA). The magnetic strength was measured using a vibrating sample magnetometer (VSM, PPMS-9, Quantum Design, San Diego, CA, USA). The specific surface area (BET) was obtained using an N_2_ adsorption-desorption apparatus (Autosorb-IQ2-MP, Quantachrome, Boynton Beach, FL, USA). Finally, using X-ray photoelectron spectroscopy (XPS, Escalab 250xi, Thermo Scientific), the binding energy of the elements contained in the materials was obtained.

### 2.5. Batch Adsorption Experiments

Before each experiment, to prevent the hydrolysis of mercury ions, 0.1 mL of HCl and 10 mL of HNO_3_ in 1000 mL pure water was used as a protective solution. A certain amount of HgCl_2_ was added to the protective solution to obtain a 1000 mg/L mercury stock solution. The effect of CoFe_2_O_4_@SiO_2_-EDTA on the adsorption of the mercury solution was studied at different pH levels. 0.01 g of the adsorbent was added into an Erlenmeyer flask containing 100 mL Hg(II) solution (*C*_0_ = 20 mg/L), and then the pH was adjusted to 2–7 using 0.1 mol/L HCl and 0.1 mol/L NaOH [34]. After shaking for 12 h, the mixture was filtered through a 0.45 µm filter and analyzed by a cold atomic absorption spectrophotometry. All experiments were performed in triplicate and then averaged. The kinetic adsorption experiment was carried out under the conditions of initial concentration *C*_0_ = 20 mg/L, pH = 7, and temperature (T) of 298 K with an adsorbent amount of 0.01 g. The contact times were 1, 3, 6, 10, 15, 30, 45, 60, 90, 120, 150, 180, 210, 240, 270, 300 and 360 min. Thermodynamic adsorption experiments were carried out at 298, 308 and 318 K with an adsorbent amount of 0.01 g, pH = 7 and *C*_0_ of 10, 20, 30, 40 and 50 mg/g.

## 3. Results and Discussion

### 3.1. Characterizations

**Figure 2:**
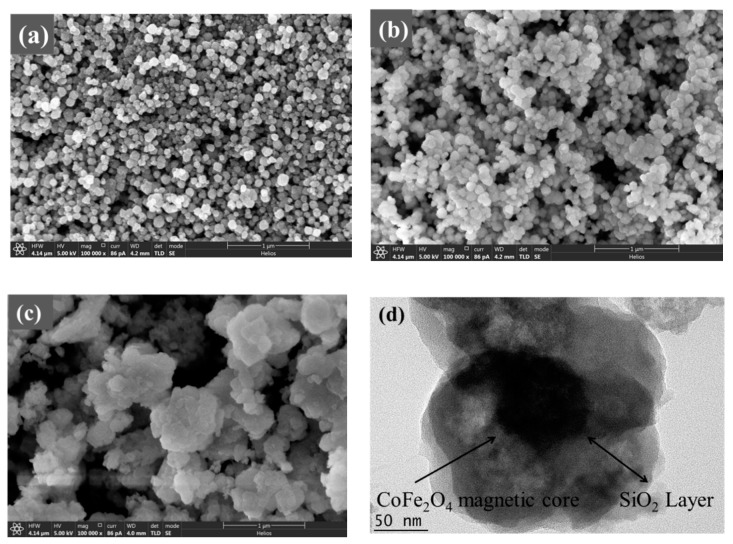
Scanning electron microscopy (SEM) images of CoFe_2_O_4_ (**a**), CoFe_2_O_4_@SiO_2_ (**b**), and CoFe_2_O_4_@SiO_2_-EDTA (**c**); transmission electron microscopy (TEM) image of CoFe_2_O_4_@SiO_2_-EDTA (**d**).

**Figure 3:**
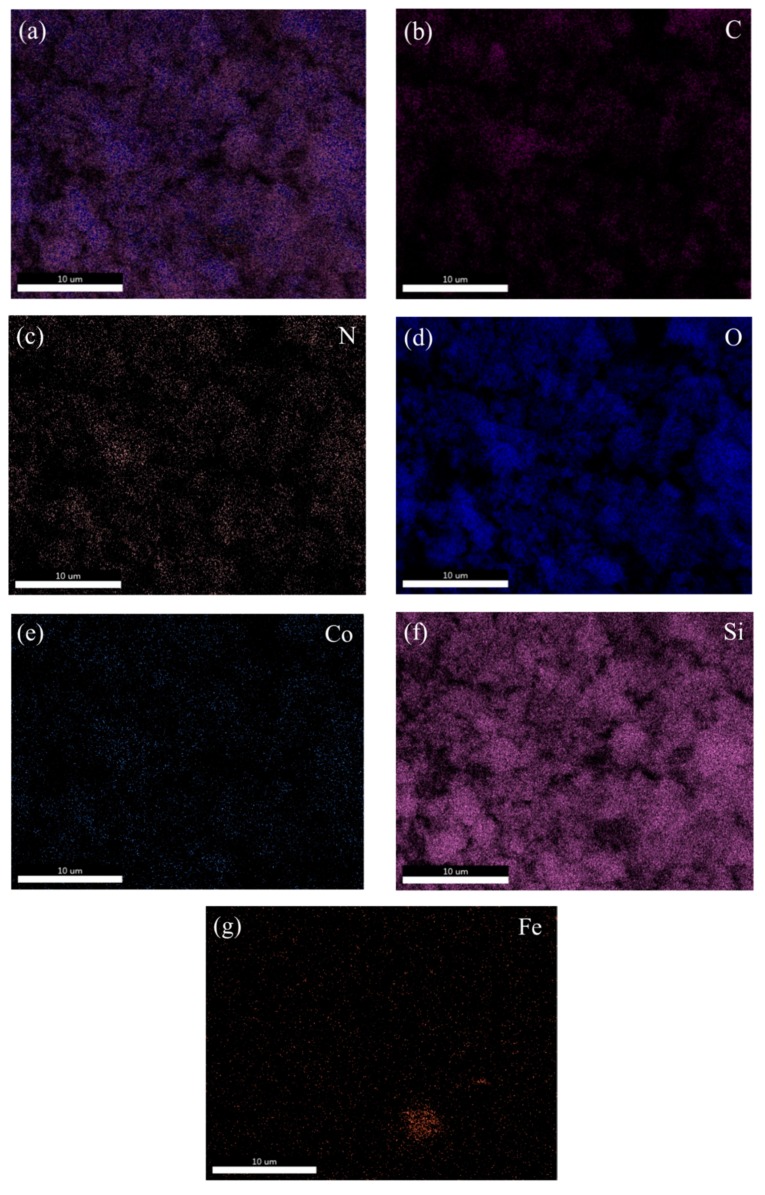
SEM image of CoFe_2_O_4_@SiO_2_-EDTA (**a**); EDS mappings of C (**b**), N (**c**), O (**d**), Co (**e**), Si (**f**) and Fe (**g**) of CoFe_2_O_4_@SiO_2_-EDTA.

From the SEM and TEM images (Figure 2 and Figure 3), it can be known that the adsorbents of CoFe_2_O_4_, CoFe_2_O_4_@SiO_2_ and CoFe_2_O_4_@SiO_2_-EDTA have the diameters of about 50–90, 90–130 and 110–200 nm. In Figure 2a, CoFe_2_O_4_ nanomaterial is irregular, and its diameter is smaller than that of CoFe_2_O_4_@SiO_2_ in Figure 2b. The SEM image clearly shows that the surface of CoFe_2_O_4_@SiO_2_ is smoother than that of CoFe_2_O_4_, which indicates that SiO_2_ is successfully supported on the surface of CoFe_2_O_4_.

After the material was modified by EDTA, its dispersibility was greatly improved with the material becoming noticeably fluffy, as shown in Figure 2c. In the TEM image of Figure 2d, Black magnetic is covered by outer silicon dioxide. Moreover, the transparent polymer coated with SiO_2_ and has a distinct core-shell structure. The energy dispersive spectrometer (EDS) mapping technology was used to analyse the elements contained in the material. Figure 3 shows the images of various elements in the material of CoFe_2_O_4_@SiO_2_-EDTA. From the image, not only the elements of Co and Fe are evenly distributed on the surface of the material, but also the other four elements of C, N, O and Si appear correspondingly and are evenly distributed, which confirms that the EDTA is successfully loaded onto the particles of the magnetic nanomaterial CoFe_2_O_4_@SiO_2_.

**Figure 4:**
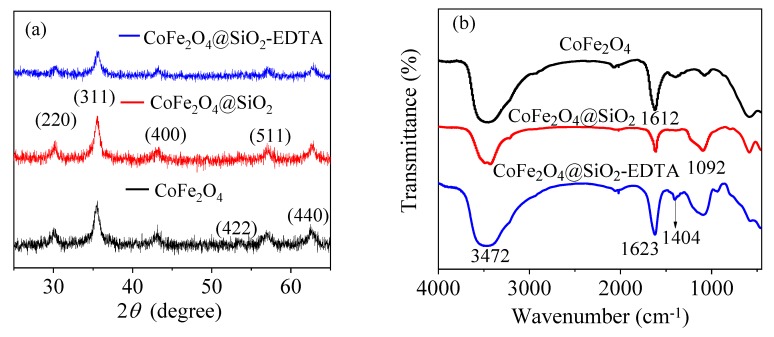
X-ray diffraction (XRD) patterns (**a**) and FT-IR spectra (**b**).

Figure 4a shows the XRD patterns of the prepared materials CoFe_2_O_4_, CoFe_2_O_4_@SiO_2_ and CoFe_2_O_4_@SiO_2_-EDTA, revealing their microcrystalline structure. The peak characteristics in the XRD pattern of CoFe_2_O_4_ are consistent with the JCPDS file (22-1086) [35]. Moreover, the diffraction peaks in the CoFe_2_O_4_@SiO_2_ and CoFe_2_O_4_@SiO_2_-EDTA are similar to those of CoFe_2_O_4_ and no other peaks are observed, indicating that the crystal structure in the material is either silicon-coated or carboxyl-functionalized. The functional groups and chemical bonds in the material were analyzed using FT-IR spectroscopy. According to Figure 4b, the broad peaks at 3472 and 1612 cm^−1^ correspond to the –OH stretching vibration peaks in the water molecules on the surface of the material [36].

In the CoFe_2_O_4_@SiO_2_ and CoFe_2_O_4_@SiO_2_-EDTA spectra, a new broad absorption peak at 1092 cm^−1^ is the stretching vibration peak of Si–O–Si [4], indicating that silica has been successfully coated on CoFe_2_O_4_. In the CoFe_2_O_4_@SiO_2_-EDTA spectrum, there is no common carboxyl peak at 1732 cm^−1^, but a new absorption peak appears at 1405 cm^−1^ [37]. This peak may be caused by EDTA modification of –COO^−^ and symmetrical stretching vibration of C–O in the group [38]. The newly emerging absorption peak at 1623 cm^−1^ may be attributed to the presence of an amide bond [39]. The results indicate that EDTA has been successfully grafted onto silica surface.

**Figure 5:**
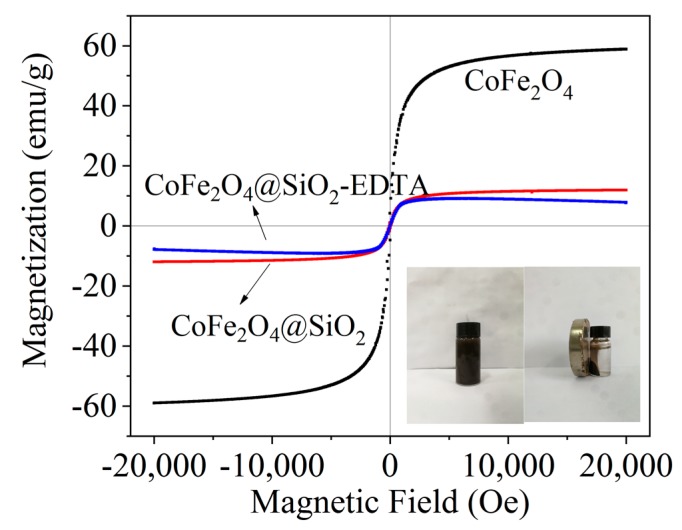
Vibrating sample magnetometer (VSM) of CoFe_2_O_4_, CoFe_2_O_4_@SiO_2_ and CoFe_2_O_4_@SiO_2_-EDTA.

Figure 5 shows the hysteresis loops of the three materials. The saturation magnetizations of CoFe_2_O_4_, CoFe_2_O_4_@SiO_2_ and CoFe_2_O_4_@SiO_2_-EDTA are 58.94, 11.94 and 7.65 emu/g, respectively. CoFe_2_O_4_ shows typical super-paramagnetism. After coated by SiO_2_, the magnetic property of CoFe_2_O_4_@SiO_2_ is obviously weakened due to the existence of silicon shell. After modified by EDTA, the thickness of the surface organic portion of CoFe_2_O_4_@SiO_2_-EDTA increases, forming a non-magnetic carboxylic acid functional layer. Although the magnetic properties are significantly reduced, the magnetic material can still be easily separated from aqueous solution. Note that the synthesis of the magnetic materials facilitates solid–liquid separation, making the entire adsorption process more convenient.

**Figure 6:**
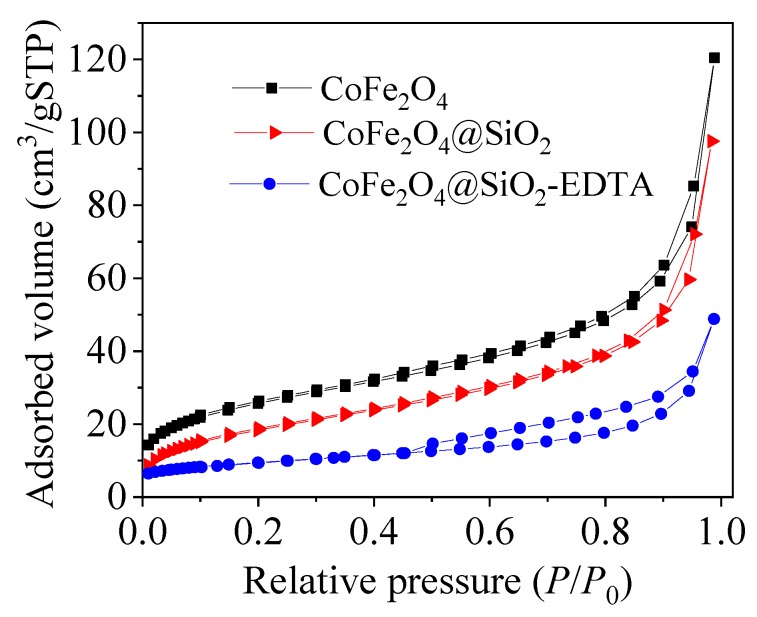
N_2_ adsorption desorption isotherms.

N_2_ desorption-desorption measurement was used to investigate the structural properties and specific surface area (BET). Figure 6 shows the adsorption-desorption isotherm curves for the three materials. The adsorption data are listed in Table 1. The BET value of CoFe_2_O_4_ decreases after the loading of the silicon shell, possibly due to the dense silica coating, resulted in a decrease of the BET value. After the modification of EDTA with the TEOS added as an active agent, the BET value slightly decreases, probably because the functionalized groups and some extra silica occupy the surface. Due to the extra fluffy silica on the surface, the pore size increases slightly (from 7.73 to 13.02 nm).

**Figure 7:**
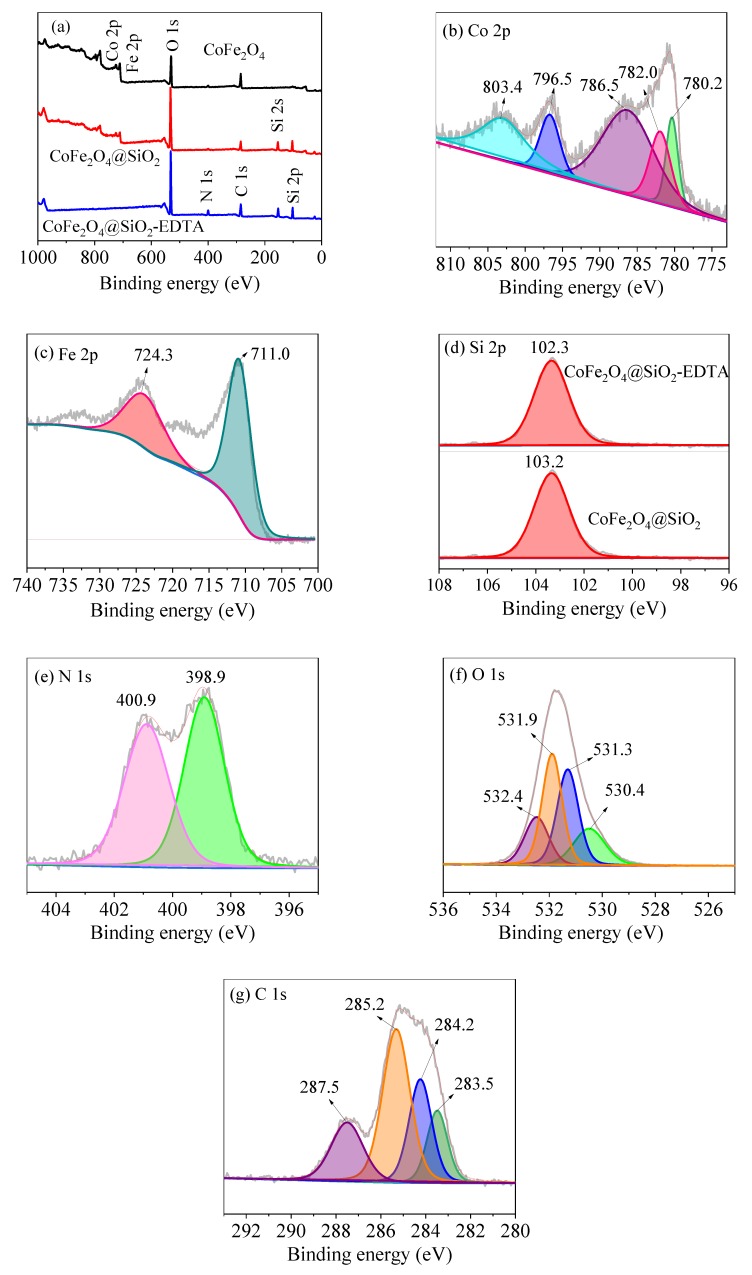
X-ray photoelectron spectroscopy (XPS) survey scan of (**a**) CoFe_2_O_4_, CoFe_2_O_4_@SiO_2_ and CoFe_2_O_4_@SiO_2_-EDTA; high-resolution scan of Co 2p (**b**), Fe 2p (**c**), Si 2p (**d**), N 1s (**e**), O 1s (**f**) and C 1s (**g**).

From the XPS spectrum of Figure 7a, the peaks of Co 2p, Fe 2p and O 1s in CoFe_2_O_4_, can be observed, and the Si 2p peak appears in the modified materials of CoFe_2_O_4_@SiO_2_ and CoFe_2_O_4_@SiO_2_. The photoelectrons of N 1s and C 1s can be attributed to the peaks that occur after EDTA-modified grafting. Moreover, high-resolution scanning of Co 2p, Fe 2p, Si 2p, N 1s, O 1s, and C 1s in the material is sequentially performed. The peaks of Co 2p_3/2_ at 780.2 and 782.0 eV are shown in Figure 7b, along with the bond energy of Co 2p_1/2_ at 796.5 eV with satellite characteristics at 786.5 and 803.4 eV, respectively.

Note that the peak at 796.5 eV is attributed to Co^3+^ oxide [40]. The primary peak of Fe 2*p* in Figure 7c at 711.0 eV is attributed to Fe^3+^, whereas that at 724.3 eV belongs to Fe^2+^ [41]. Figure 7d shows the change of Si before and after modification with EDTA. The peak of Si 2p appears at 103.2 eV before modification and at 102.3 eV after modification. The significant decrease of the Si 2p peak after modification can be explained by the successful grafting of EDTA. Figure 7e shows a scan of N 1s with binding energies of C–N and N–H in the N element at 400.9 and 398.9 eV, respectively.

Figure 7f shows a high-resolution scan of O 1*s*, which contains four different binding energy values: 532.4 and 531.9 eV correspond to carbon-based O–C=O and Si–O/C–O [42], respectively, whereas 531.3 eV corresponds to the surface hydroxyl group O–H, and 530.4 eV corresponds to Co–O/Fe–O [40,43]. In the C 1s scan of Figure 7g, the four different binding energy values observed at 283.5, 284.2, 285.2 and 287.5 eV correspond to C in C–Si, C–H, C–C/C–N and O–C=O, respectively. Thus, through XPS characterization, SiO_2_ is supported onto CoFe_2_O_4_ and EDTA is successfully grafted [44].

**Figure 8:**
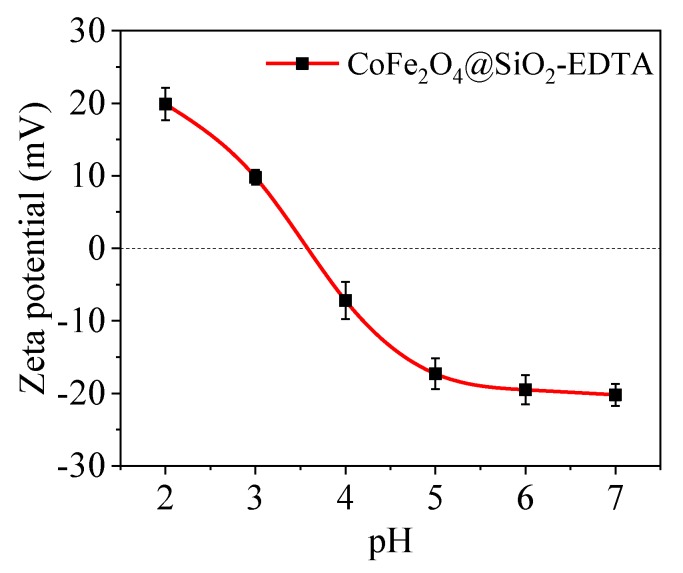
Zeta potential curves of CoFe_2_O_4_@SiO_2_-EDTA.

As shown in Figure 8, the equipotential (pH_iep_) of the adsorbent is ~3.6; therefore, the material’s surface is negatively charged at pH ≥ 3.6. According to the experimental results, the maximum adsorption capacity of CoFe_2_O_4_@SiO_2_-EDTA at pH 7 is closely related to the electrostatic interaction between the surface negative charge and the Hg^2+^ cation. When pH is lower than pH_iep_, the decrease in the adsorption capacity of CoFe_2_O_4_@SiO_2_-EDTA can be attributed to the positive charge on the surface of the material and the repulsive force between Hg^2+^ and the adsorbent.

### 3.2. Adsorption Performance

#### 3.2.1. Effect of EDTA Addition Amount

**Figure 9:**
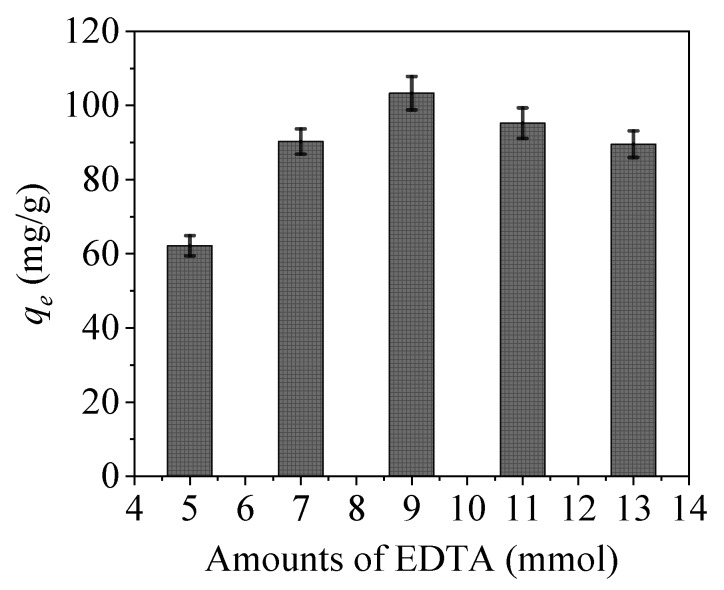
Effect of EDTA addition on (*C*_0_ = 20mg/L, pH = 7, dosage = 0.01 g, *t* = 6 h and *T* = 298 K).

Generally, the amounts of EDTA and the added intermediate crosslinking agent of APTES significantly affect the material properties (Figure 9). The adsorption amount of CoFe_2_O_4_@SiO_2_-EDTA increases with increasing amounts of these additives. When the EDTA amount is 9 mmol, the adsorbent has a maximum adsorption capacity of *q*_e_ of 103.3 mg/g. However, when the amount is less than 9 mmol, then the active site of the functional group remains on the surface of CoFe_2_O_4_@SiO_2_ so that the adsorption amount is reduced. Furthermore, when the amount added is greater than 9 mmol, the excess amount of EDTA will self-condense in the material, resulting in the coverage of the active groups, whereby the grafting ratio is lowered and the adsorption is decreased. Therefore, for subsequent experiments, EDTA amount of 9 mmol was selected for the subsequent study.

#### 3.2.2. Effect of pH

**Figure 10:**
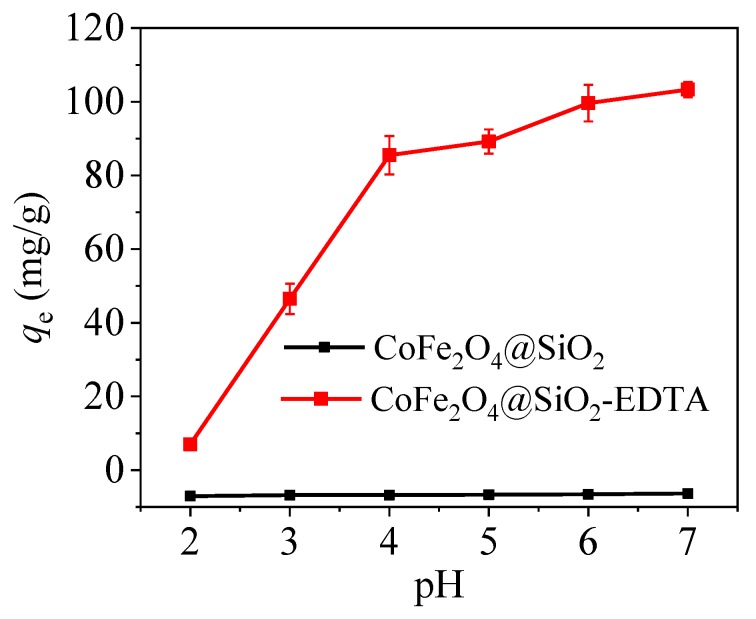
Effect of pH on the removal of Hg(II) by CoFe_2_O_4_@SiO_2_-EDTA as adsorbent (dosage = 0.1 g/L, *C*_0_ = 20 mg/L, *t* = 6 h, *T* = 298 K).

As an important factor in adsorption, pH value directly affects the surface charge of the adsorbent surface, the existence morphology of heavy metal mercury ions and the stability of the interaction between the functional groups and the mercury ions. According to the data presented in Figure 10, with increasing pH value, the adsorption capacity for mercury ions with CoFe_2_O_4_@SiO_2_ is always low, but that of CoFe_2_O_4_@SiO_2_-EDTA quickly increases. At pH of 7, CoFe_2_O_4_@SiO_2_-EDTA has a maximum capacity of 103.3 mg/g for Hg(II). When the pH is alkaline, there is no need for detection because the carboxylic acid in EDTA is neutralized under alkaline conditions, and the adsorption capacity is considerably affected. Furthermore, a high pH value is not conducive for practical applications. At low pH, the solution contains a large amount of H^+^, the EDTA functional group is completely protonated and electrostatic repulsion occurs with the mercury ion, and EDTA exists in the form of –COOH while there is shortage of coordination sites, which weakens the complexation between functional groups and Hg^2+^, and leads to low adsorption capacity.

As pH value is increased, the H^+^ in the carboxylic acid gradually dissociates from the functional group, Until the EDTA functional group is completely deprotonated and exists in the form of –COO^−^, and the competitiveness of H^+^ becomes weaker as its concentration decreases, thereby enhancing the affinity of adsorbent towards Hg^2+^. Thus, the adsorption of Hg(II) by CoFe_2_O_4_@SiO_2_-EDTA is mainly through ion exchange and chelation [45].

#### 3.2.3. Effect of Dosage

**Figure 11:**
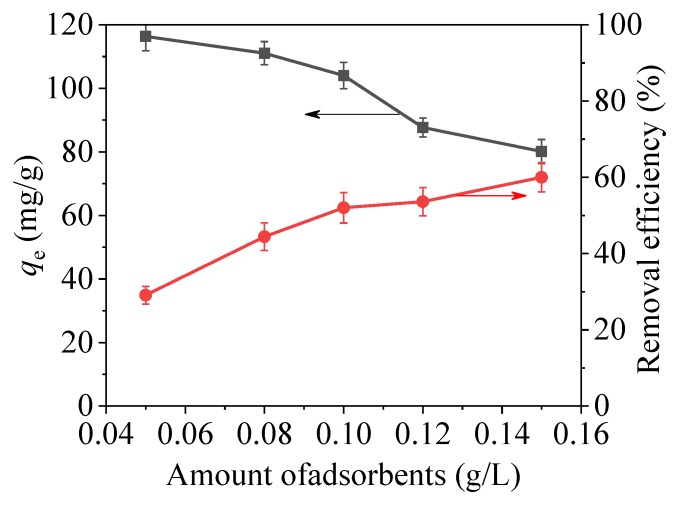
Effects of dosage with CoFe_2_O_4_@SiO_2_-EDTA as adsorbent (pH = 7, *C*_0_ = 20 mg/L, *t* = 6 h, *T* = 298 K).

As shown in Figure 11, the effect of adsorption capacity of mercury ions was researched with different dosage of CoFe_2_O_4_@SiO_2_-EDTA under the same conditions. As the dosage is increased, the adsorption capacity for Hg(II) is rapidly decreased, and the adsorption efficiency is rapidly increased. Thus, the increase of dosage provides more active sites for the combination of –COOH and Hg(II), resulting in a high removal rate. However, the effective utilization between the material and Hg(II) is reduced, thereby decreasing the adsorption capacity.

### 3.3. Adsorption Kinetics

**Figure 12:**
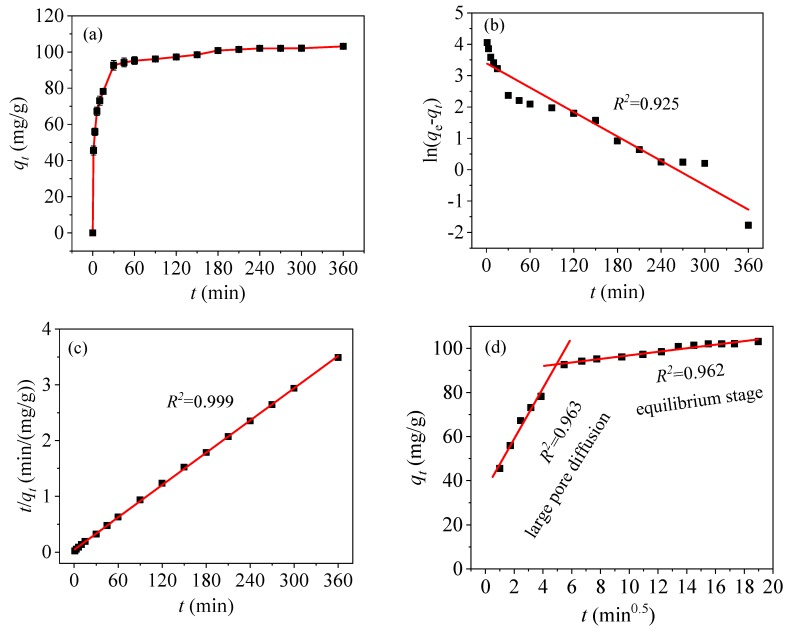
Kinetics of *q_t_* vs. *t* (**a**), pseudo-first-order (**b**), pseudo-second-order (**c**), and intra-particle diffusion models (**d**).

Figure 12a shows the adsorption curve of Hg(II) as a function of time. Rapid adsorption occurs in the first 30 min, and the adsorption capacity reaches 92.63 mg/g. The –COOH groups in the CoFe_2_O_4_@SiO_2_-EDTA provides rich active sites, thus leading to the complexation and rapid interaction of Hg(II) in the first 30 min [46]. Then adsorption begins to increase slowly and approaches maximum at 360 min with a capacity of 103.13 mg/g, owing to the presence of a large number of –COOH groups.

To explore the adsorption behavior of CoFe_2_O_4_@SiO_2_-EDTA in detail, three types of kinetic models were used to fit the experimental data, including a pseudo-first-order kinetic model (Equation (1)), pseudo-second-order kinetic model (Equation (2)) and intra-particle diffusion model (Equation (3)):

(1) $$\ln=(q_{e}-q_{t})=\ln q_{e}-k_{1}t$$

(2) $$\frac{t}{q_{t}}=\frac{1}{{q_{e}}^{2}k_{2}}+\frac{t}{q_{e}}$$

(3) $$q_{t}=k_{d}t^{0.5}+C$$

where *q_e_* (mg/g) is the equilibrium adsorption capacity; *q_t_* (mg/g) is the adsorption capacity at time *t* (min); *k*_1_ and *k*_2_ are the adsorption rate constants; *k_d_* is the intraparticle diffusion rate constant and *C* (mg/g) is the boundary layer thickness.

Based on the fitting results of the above three kinetic models and Table 2, from the linear fitting in Figure 12b,c, the regression coefficient of the pseudo-first-order model (*R*^2^ = 0.925) is less than that of the pseudo-second-order model (*R*^2^ = 0.999). From the pseudo-first-order fitting diagram, it can be observed that the distributions of points are scattered and the fitting is poor. Kinetic adsorption is not accounted for in the pseudo-first-order model. However, in the pseudo-second-order model, the fitting is extremely high [47]. Moreover, the fitting value of *q_e_*_,cal_ (103.62 mg/g) is closer to the experimental value of *q_e_*_,exp_ (103.13 mg/g). Thus, it can be concluded that the adsorption of Hg(II) in an aqueous solution with CoFe_2_O_4_@SiO_2_-EDTA can be explained by the pseudo-second-order kinetic model, and the adsorption process is subjected to chemisorption [45]. This behavior refers to the complexation between the adsorbent and mercury ions and forms a chemical bond during the process of adsorption [48].

Figure 12d shows a fitting plot of the intra-particle diffusion model. The adsorption process is divided into two stages. The first-stage is large pore diffusion, corresponding to fast adsorption and high *k_d_*_1_ values. Finally, the second stage is equilibrium adsorption, corresponding to low adsorption rates and *k_d_*_2_ values. However, in the linear fitting, the straight line does not pass through the origin, indicating that intra-particle diffusion is not the only factor affecting the adsorption rate of mercury.

### 3.4. Adsorption Isotherms

**Figure 13:**
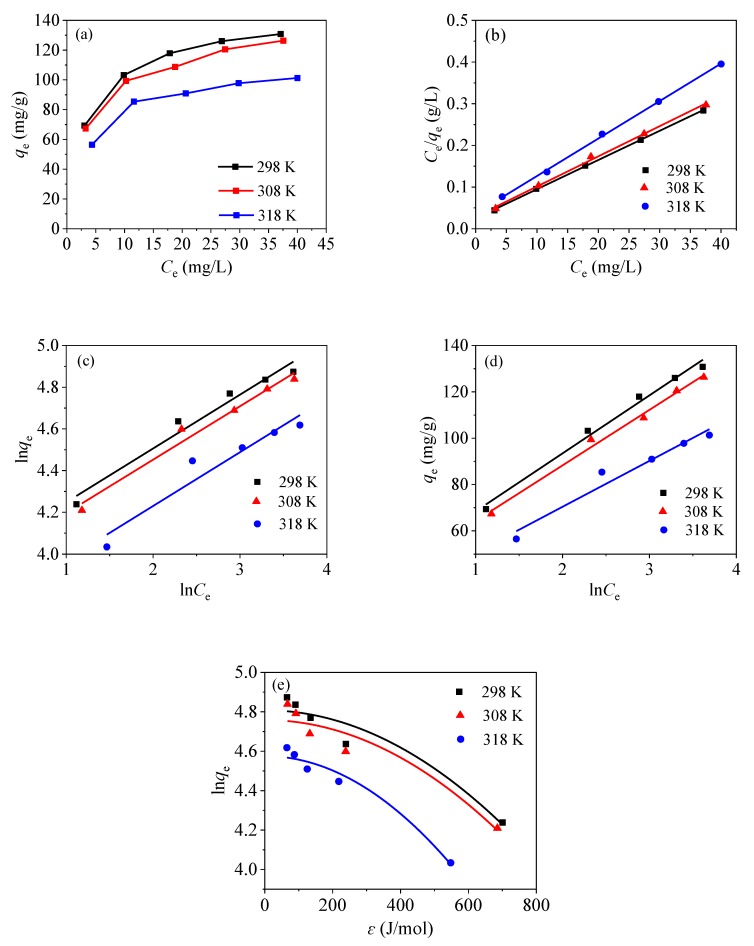
Adsorption isotherm of CoFe_2_O_4_@SiO_2_-EDTA on Hg(II) (**a**), Langmuir (**b**), Freundlich (**c**), Temkin (**d**), and Dubinin–Radushkevich (**e**).

Figure 13a shows the adsorption of mercury ions in an aqueous solution with the adsorbent CoFe_2_O_4_@SiO_2_-EDTA at different temperatures. According to the above data, the material has an optimal adsorption effect at 298 K and pH of 7. As the temperature increases, the adsorption curve gradually decreases, indicating that the material is more suitable for using under room temperature conditions. Note that the adsorption isotherm is important for optimizing the using of the adsorbent, as it can be used to evaluate the adsorption capacity of the adsorbent and describe how the adsorbent interacts with the adsorbates.

The Langmuir isotherm model (Equation (4)), Freundlich isotherm model (Equation (5)), Temkin isotherm model (Equation (6)) and Dubinin–Radushkevich isotherm model (Equations (7)–(9)) can be obtained using the following equations:

(4) $$\frac{C_{e}}{q_{e}}=\frac{C_{e}}{Q_{m}}+\frac{1}{Q_{m}K_{L}}$$

(5) $$\ln q_{e}=\ln K_{F}=\frac{1}{n}\ln C_{e}$$

(6) $$q_{e}=\frac{RT}{b_{T}}\ln K_{T}C_{e}$$

(7) $$\ln q_{e}=\ln q_{m}-\beta \varepsilon^{2}$$

(8) $$\varepsilon =RT\ln(1+\frac{1}{C_{e}})$$

(9) $$E=\frac{1}{\sqrt{2\beta }}$$

where *Q_m_* (mg/g) is the saturated adsorption capacity; *C_e_* (mg/L) is the equilibrium concentration; *K_L_* (L/mg) is the Langmuir adsorption equilibrium constant; *K_F_* and *K_T_* are all constants. *R* is the gas constant (8.314 J/mol/K); *T* (K) is the temperature, *ɛ* is the Polanyi potential energy and *β* is a constant related to average adsorption energy *E* (kJ/mol). The separation constant *R_L_* can be used to describe the adsorption characteristics of the Langmuir adsorption isotherm model using Equation (10).

(10) $$R_{L}=\frac{1}{1+K_{L}C_{0}}$$

where *R_L_* is a separation constant, which is dimensionless, and *C*_0_ (mg/L) is the initial concentration.

The fitting maps for the Langmuir, Freundlich, Temkin and Dubinin–Radushkevich isothermal models are shown in Figure 13b–e, and the corresponding data are shown in Table 3. According to the fitting data, the three *R*^2^ of the Langmuir model are higher than those of the Freundlich model under different temperatures, indicating that the adsorption process is a single molecule adsorption process [34]. Therefore, the adsorbent exhibits a chemisorption behavior [49]. This conclusion is consistent with the kinetic model. From Table 3, it can be observed that the maximum adsorption capacities of the materials fitted by the Langmuir isothermal model are 143.85, 138.12 and 111.23 mg/g, respectively, when the temperature is 298, 308 and 318 K. Therefore, as the temperature increases, the adsorption capacity gradually decreases.

Moreover, the value of *R_L_* is between 0 and 1, which indicates that the divalent mercury ions are easily adsorbed by the material. In the Freundlich isotherm model, the *1/n* values are also between 0 and 1, indicating that the adsorption process is proceeding in a favourable direction. The relatively high *R*^2^ values and the *K_T_* values in the Temkin isotherm model at three temperatures indicate a strong interaction between CoFe_2_O_4_@SiO_2_-EDTA and mercury ions; while the increasing *b_T_* values with the rising temperature indicate that the adsorption capacity is gradually decreasing. The result confirms that the high temperature does not favour the reaction.

In the Dubinin–Radushkevich model, when the value of the mean free energy *E* < 8 kJ/mol, the reaction belongs to physical adsorption; however, when the values of *E* is between 8 and 16 kJ/mol, ion exchange takes place [50], and the chemisorption mechanism is functioning when the values of *E* is exceeds 16 kJ/mol. As the average free energy *E* of the three adsorbents is over 16 kJ/mol, chemisorption is involved in the adsorption process, which is consistent with the abovementioned conclusions.

As shown in Table 4, CoFe_2_O_4_@SiO_2_-EDTA had a smaller specific surface area and a higher adsorption capacity, which is better than most adsorbents.

### 3.5. Thermodynamics

The nature and mechanism of the adsorption process can be analyzed by thermodynamics. The standard Gibbs free energy (Δ*G*^0^, kJ/mol), enthalpy change (Δ*H*^0^, kJ/mol) and entropy change (Δ*S*^0^, kJ/mol/K) are represented by Equations (11) and (12).

(11) $$\Delta G^{0}=-RT\ln K_{d}$$

(12) $$\ln K_{d}=\frac{\Delta S^{0}}{R}-\frac{\Delta H^{0}}{RT}$$

where *K_d_* is equilibrium constant.

**Figure 14:**
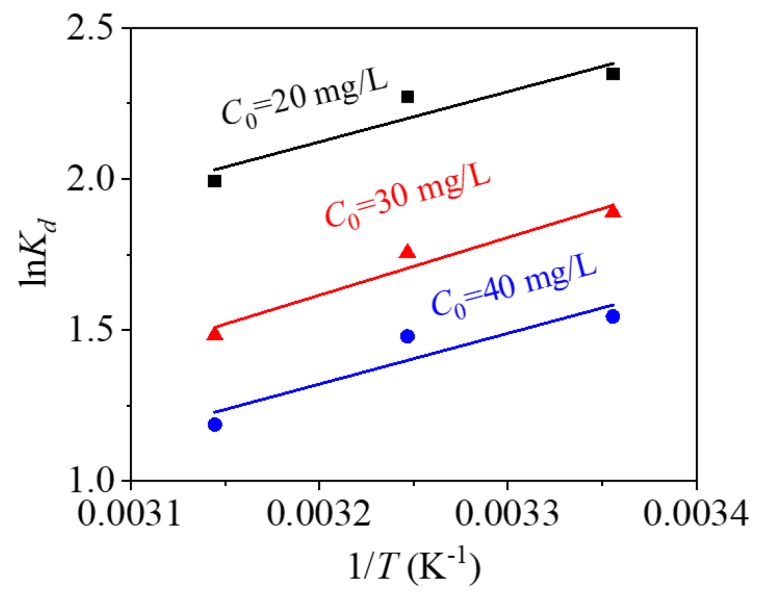
Thermodynamic fitting of the adsorption of Hg(II) by CoFe_2_O_4_@SiO_2_-EDTA.

From thermodynamic fitting of Figure 14 and the thermodynamic fitting parameters of Table 5, the Δ*H*^0^ values are negative at three different mercury ion concentrations, indicating that the process of adsorbing Hg^2+^ by CoFe_2_O_4_@SiO_2_-EDTA is exothermic. All of the Δ*S*^0^ are negative, indicating that the order of the solid–liquid interface increases during the process of adsorption [36], Δ*G*^0^ are also negative, indicating a spontaneous adsorption. As temperature increases, Δ*G*^0^ gradually decreases, indicating that high temperature is not conducive to the progress of the adsorption process. Based on the fitting results and data, the adsorption of mercury ions by the CoFe_2_O_4_@SiO_2_-EDTA is a spontaneous exothermic reaction.

### 3.6. Reusability

**Figure 15:**
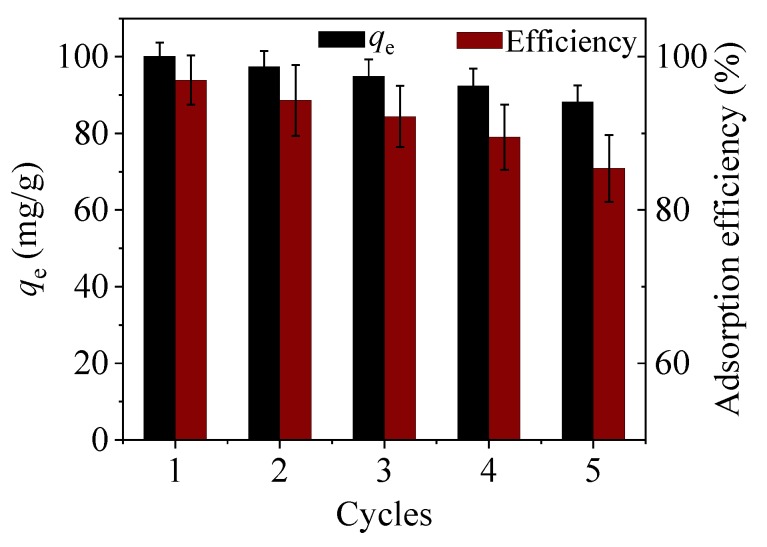
Regeneration cycle of CoFe_2_O_4_@SiO_2_-EDTA.

To examine the potential ability of CoFe_2_O_4_@SiO_2_-EDTA in practical applications, the regeneration of the adsorbent was evaluated by repeatedly treating CoFe_2_O_4_@SiO_2_-EDTA-Hg with 0.1 mol/L HCl as a regenerant [45]. The mixture is stirred for 4 h under acidic conditions. Figure 15 shows that the adsorption capacity of the material decreases after each regeneration cycle. After three cycles, >90% of the adsorbed Hg(II) still remains; the adsorption capacity decreases by ~14.5% after five cycles, which was a favourable result. Thus, the regeneration studies show that the adsorbent of CoFe_2_O_4_@SiO_2_-EDTA has a good potential application in actual water treatment [34].

### 3.7. Mechanism Speculation

**Figure 16:**
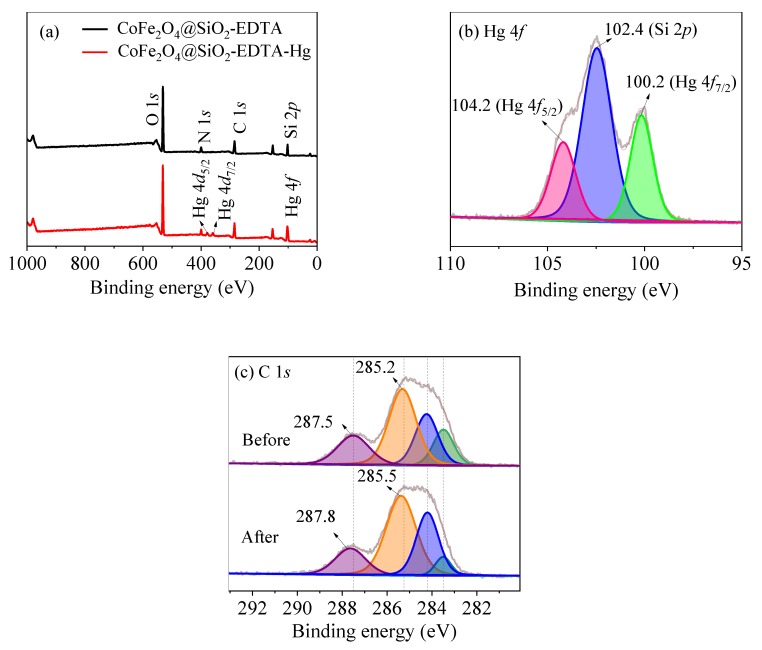
XPS spectra of survey scan of (**a**) and high-resolution scan of Hg 4*f* (**b**) and C 1*s* (**c**).

To study the adsorption mechanism of CoFe_2_O_4_@SiO_2_-EDTA towards Hg(II) and analysis was carried out by using XPS technology. It can be seen from Figure 16a, a new peak of Hg appears after adsorption. The peaks of Hg 4*f*_5/2_ at 104.2 eV and of Hg 4*f*_7/2_ at 100.2 eV (Figure 16b) are attributed to the adsorbed HgCl_2_ [57]. It clearly shows that Hg is successfully adsorbed by CoFe_2_O_4_@SiO_2_-EDTA. In Figure 16c, the O–C=O and C–N/C–C are transferred to 285.5 and 287.8 eV, respectively, compared to the spectrum before adsorption [44]. The reason is the formation of coordination bonds between the carboxylic acid and Hg(II) on the surface of the material. Therefore, XPS spectra indirectly confirmed that a stable complex was formed by chelation between CoFe_2_O_4_@SiO_2_-EDTA and mercury ions during the process of adsorption.

Compared the morphology of the different types of Hg(II) in aqueous solution and the binding sites that can be utilized, it can be observed that the adsorption effect is not very different. When the solution contains Cl^−^, the form of Hg(II) in the solution, such as HgCl_2_, HgCl^+^ and Hg(OH)_2_, changes with pH value [16]. Under strongly acidic conditions, the surface of the adsorbent is negatively charged, and the EDTA functional group on the surface of the adsorbent is protonated, which limits carboxyl binding to Hg^2+^.

However, the surface of the adsorbent is positively charged with the increase of pH, and the presence of EDTA exhibits a strong chelating ability to metal ions. Mercury ions in the solution gradually form hydroxy mercury (HgOH and Hg(OH)_2_) [58] and interaction with the adsorbent occurs, there is no static repulsion, and the adsorption effect quickly reaches the maximum value.

**Figure 17:**
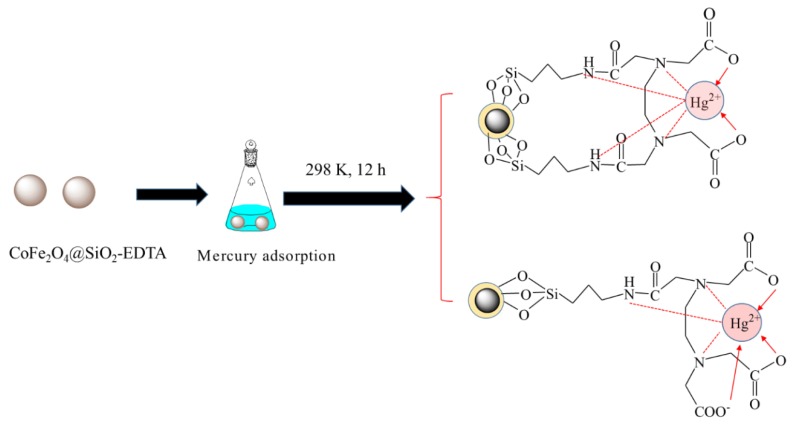
Possible mechanism of adsorption of mercury.

The adsorption mechanism of mercury by CoFe_2_O_4_@SiO_2_-EDTA is shown in Figure 17. A complex with cyclic structure is formed from the central COO^−^ ions and the N-based ligand under the chelation. During the process of adsorption, there are two situations in Figure 17. By EDTA-functionalized, the adsorbent forms a symmetric ligand or contain a free carboxyl group. Under these conditions, a stable complex is often formed by H_2_O and another bond, but H_2_O is usually replaced by an imine group on CoFe_2_O_4_@SiO_2_-EDTA to form a more stable six-position complex [34,37]. Understanding the complex mechanisms in the adsorption process requires collecting data to determine rates, steps and related parameters. Here, the removal of Hg(II) by CoFe_2_O_4_@SiO_2_-EDTA is evaluated considering different adsorption kinetics and adsorption isotherm models. The adsorption process of metal ions can be divided into two steps [58].

**Figure 18:**
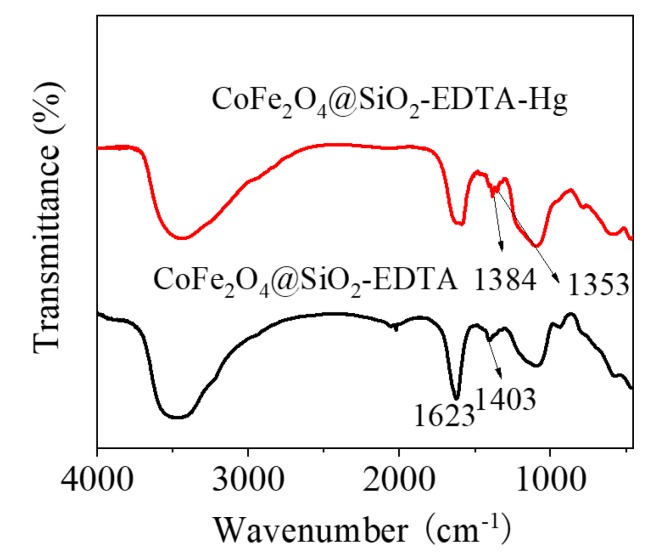
FT-IR spectra before and after adsorption by CoFe_2_O_4_@SiO_2_-EDTA.

The first process involves the adsorption of Hg ions to the active sites on the surface of the material through functional groups, during which physical or chemical adsorption occurs. The experimental data show excellent adaptation in the pseudo-second-order kinetic and the Langmuir isotherm models [59]. The homogeneous active site on the adsorbent CoFe_2_O_4_@SiO_2_-EDTA is a single molecule adsorption process for mercury ions. Therefore, chemical adsorption is the driving force in the rate-control step of the adsorption process. Moreover, the value of the mean free energy *E* in the Dubinin–Radushkevich isothermal model confirms this. Furthermore, this illustrates the complexation between the metal ions and the active sites on the adsorbent during the process of adsorption. A stable complex is formed by chelation between a carboxylic acid and Hg^2+^. To further understand the adsorption mechanism, the FT-IR spectra after adsorption show that the tensile vibration at 1646 cm^−1^ is lost. The result indicates that the carbonyl group of EDTA interacts with Hg(II). It can be seen from Figure 18, with the disappearance of the vibrational peak of carboxylic acid at 1403 cm^−1^ after adsorption, the peaks at 1353 and 1384 cm^−1^ are attributed to the adsorption of the Hg carboxylic acid [38]. This group forms a new chemical bond with Hg, resulting in a shift in the peak. The change of the peak value in the FT-IR spectrum also indirectly confirms that the adsorption process of mercury ions in aqueous solution by CoFe_2_O_4_@SiO_2_-EDTA is chemical adsorption. Finally, Figure 15 and Figure 16 show that the CoFe_2_O_4_@SiO_2_-EDTA material before and after adsorption does not significantly change, thereby indicating that the material has good hydrothermal stability.

## 4. Conclusions

In this study, a core-shell structure of CoFe_2_O_4_@SiO_2_ is successfully functionalized with EDTA by a safe, mild and easy hydrothermal method. The as-prepared material has a saturation magnetic strength of 7.65 emu/g. Moreover, the material has a maximum adsorption capacity of 103.3 mg/g for metal Hg(II) at pH 7, and rapid separation of the adsorbent from the solution by magnetism. The adsorption process has excellent correlation with pseudo-second-order kinetics and the Langmuir isotherm model; moreover, it is a single-layer adsorption and a spontaneous exothermic reaction. A stable complex is formed between the EDTA functional group and the Hg(II) ion by chelation, and the chemical reaction is the key to the rate control step of the adsorption process. To summarize, EDTA-Functionalized Magnetic CoFe_2_O_4_@SiO_2_ Nanomaterial has good hydrothermal stability, recyclability and fully biodegradable properties, indicating that it is a potential adsorbent for removing heavy metals from water in practical applications.

## Figures and Tables

**Table 1:** N_2_ adsorption desorption isothermal data of the as-prepared materials.

Samples	BET Values (m^2^/g)	Total Pore Volumes (cm^3^/g)	Pore Diameters (nm)
CoFe_2_O_4_	91.85	0.176	7.67
CoFe_2_O_4_@SiO_2_	83.02	0.160	7.73
CoFe_2_O_4_@SiO_2_-EDTA	20.09	0.065	13.02

**Table 2:** Adsorption kinetic parameters.

**Pseudo-First-Order**				**Pseudo-Second-Order**		
***q_e_*_,exp_**	***q_e_*_,cal_**	***k*_1_**	***R*^2^**	***q_e_*_,cal_**	***k*_2_**	***R*^2^**
103.13	29.80	0.013	0.925	103.62	0.009	0.999
**Intra-Particle Diffusion**						
***k_d_*_1_**		***C*_1_**	***R*^2^**	***k_d_*_2_**	***C*_2_**	***R*^2^**
11.507		35.89	0.963	0.807	88.77	0.962

**Table 3:** Adsorption isotherm model parameters.

***T* (K)**	**Langmuir Model**				**Freundlich Model**		
	***Q_m_* (mg/g)**	***K_L_* (L/mg)**	***R*^2^**	***R_L_***	***1/n***	***K_F_***	***R*^2^**
298	142.85	0.2811	0.999	0.066	0.258	54.04	0.957
308	138.12	0.2491	0.996	0.074	0.256	51.39	0.968
318	111.23	0.2454	0.998	0.075	0.258	41.01	0.903
***T* (K)**	**Temkin Model**				**Dubinin–Radushkevich Model**		
	***b_T_***		***K_T_***	***R*^2^**	***Q_max_* (mg/g)**	***E* (KJ/mol)**	***R*^2^**
298	98.95		5.65	0.986	122.48	20.55	0.911
308	106.83		5.37	0.989	116.56	20.47	0.892
318	133.38		4.71	0.943	97.06	16.53	0.963

**Table 4:** Comparison of adsorption capacities for Hg(II) onto different absorbents.

Adsorbents	BET (m^2^/g)	pH	Fitting Models	*Q_m_* (mg/g)	Ref.
M-ATP	116.56	4	Langmuir	90.0	[51]
Cys-d-FeOOH	34	7	Langmuir	35.0	[52]
MPTS-CNTs/Fe_3_O_4_	97	6	Langmuir	65.5	[53]
Bi_2_O_4_/ZnO	–	7	Langmuir	60.0	[54]
M. pyrifera	–	5	Langmuir	80.0	[55]
o-benzenedithiol-modified cellulose	–	6	Langmuir	86.0	[56]
CoFe_2_O_4_@SiO_2_-EDTA	20.09	7	Langmuir	103.3	This work

**Table 5:** Adsorption kinetic parameters.

*C*_0_ (mg/L)	Δ*H*^0^ (KJ/mol)	Δ*S*^0^ (J/mol/K)	Δ*G*^0^ (KJ/mol)		
			298 K	308 K	318 K
20	−13.83	−26.61	−5.814	−5.691	−5.270
30	−15.89	−37.42	−4.678	−4.495	−3.921
40	−19.98	−33.75	−3.826	−3.787	−3.138
